# Monte Carlo modeling of radiation dose from radiation therapy with superficial x‐rays

**DOI:** 10.1002/acm2.70062

**Published:** 2025-03-04

**Authors:** Reham Barghash, Tiffany W. Martin, Amber R. Prebble, Del Leary

**Affiliations:** ^1^ Environmental and Radiological Health Sciences Colorado State University Fort Collins USA; ^2^ College of Applied Medical Sciences King Saud bin Abdulaziz University for Health Sciences Riyadh Saudi Arabia; ^3^ King Abdullah International Medical Research Center King Saud bin Abdulaziz University for Health Sciences Riyadh Saudi Arabia; ^4^ Department of Clinical Sciences Colorado State University Fort Collins USA; ^5^ Veterinary Teaching Hospital Colorado State University Fort Collins USA

**Keywords:** bone dose, EGSnrc, HVL, Monte Carlo simulations, PDD, photoelectric effect, radiotherapy, superficial x‐rays

## Abstract

**Introduction:**

Superficial x‐rays (50–100 kVp) are used for treating non‐melanoma skin cancer and intraoperative radiation therapy (IORT). At these energies, the photoelectric effect significantly increases absorbed dose to bone compared to soft tissue.

**Methods:**

We used EGSnrc MC simulations to investigate bone dose enhancement during radiotherapy with the Sensus SRT‐100 machine. Simulated beams were validated against laboratory measurements and compared to a commercial treatment planning system (SmART‐ATP). Transmission simulations indirectly predicted bone dosage. Simulated beams were utilized as a mock treatment plan from a human cone‐beam computed tomography (CBCT) image.

**Results:**

EGSnrc accurately modeled the Sensus SRT‐100 beams (100, 70, and 50 kVp) with a root mean square error (RMSE) of percentage depth dose ratios between Monte Carlo predictions and lab measurements of 1.66, 0.47, and 0.99, respectively. PDDs from simulations of a water phantom with bone slabs showed peak doses at water–bone interfaces relative to surface doses. At 0.3 cm depth bone slab, doses reached 410%, 490%, and 510% for 50, 70, and 100 kVp, respectively. At 1.5 cm depth, doses were 140%, 215%, and 270%. At 2.5 cm depth, peak doses were 74%, 130%, and 170% for 50, 70, and 100 kVp, respectively. A simulated treatment plan (4 Gy surface dose) using a CBCT of a human head predicted the dose to the skull to be around 20, 19, and 15 Gy for the 100, 70, and 50 kVp beams, respectively.

**Conclusions:**

The study demonstrated EGSnrc's efficiency in conjunction with SmART‐ATP for treatment planning. MC simulations effectively quantified bone dose enhancement during superficial x‐ray radiotherapy, highlighting its importance in treatment planning and dose calculations. Clinicians should consider measuring bone depth prior to treatment to avoid excessive bone dose.

## 1 INTRODUCTION

Intraoperative radiation therapy (IORT) represents an innovative approach to cancer treatment, facilitating the precise delivery of high‐dose radiation to the tumor site during surgery. Compared to conventional radiation therapy, IORT offers the advantage of direct visualization of the tumor bed, enabling the temporary displacement or shielding of adjacent organs from radiation exposure. This multidisciplinary treatment strategy, often combined with external beam radiation and chemotherapy, is particularly valuable in managing locally advanced and recurrent cancers.^1^, ^2^, ^3^, ^4^, ^5^

Various radiation sources are employed in IORT, including high‐dose‐rate (HDR) brachytherapy, electrons with energies ranging from 2 to 9 MeV, orthovoltage x‐rays (100–500 kVp), and superficial x‐rays (50–100 kVp).^6^, ^7^, ^8^, ^9^, ^10^ Superficial x‐ray machines deliver a superficial radiation dosage that penetrates to shallow depths in the body, typically a few millimeters to a few centimeters. This superficial radiation therapy technique is typically used to treat non‐melanoma skin cancers (such as basal cell carcinoma and squamous cell carcinoma), as well as the non‐malignant tumor cells that form keloids.^11^, ^12^

A critical consideration in superficial x‐ray‐based IORT is the variation in radiation absorption between different tissues. At low x‐ray energies, the photoelectric effect dominates. The photoelectric effect involves the complete transfer of a photon's energy to an orbital electron. Notably, bone absorbs a greater fraction of low energy photons compared to soft tissue due to its higher effective atomic number. The probability of photoelectric absorption is roughly proportional to (Z/E),^3^ where Z is the tissue atomic number and E is the photon energy.^14^ The atomic number of bone tissues is 20, while soft tissue primarily consists of carbon (with atomic number 6), hydrogen (with atomic number 1), and oxygen (with atomic number 8).^15^ The equivalent dose (which accounts for biological effectiveness) from the absorbed dose of different tissues can be estimated by calculating the f‐factor.^16^ The f‐factor, also known as the radiation weighting factor, is a conversion factor used in radiation protection and dosimetry. It serves to quantify the biological effectiveness of different types of ionizing radiation relative to reference radiation, typically x‐rays or gamma rays. It links the ionizing radiation exposure (SI unit: coulombs per kilogram) with the absorbed dose of that radiation (gray) that reflects the varying biological effects of different types of radiation.^17^ The effective atomic number (Z) of different materials (bone, muscle, and air) absorbs radiation differently due to their atomic composition.^18^ By applying appropriate f‐factors, radiation professionals can better assess and manage tissue‐specific risks associated with exposure to various forms of ionizing radiation.^19^ The effective Z of air and soft tissue are nearly the same, resulting in a relative f‐factor for these tissues with superficial x‐rays to be approximately 1. However, because of bone's greater effective Z, compared to soft tissue, it has an f‐factor of around 4.25.^20^, ^21^ This poses a challenge when predicting the dose to bone ratio within the radiation beam during superficial x‐ray‐based IORT procedures.^22^

The dosimetry of superficial radiotherapy, particularly when bone is involved, has been studied using both experimental and Monte Carlo (MC) methods. Chow and Grigorov (2012) used EGSnrc to assess the impact of a 1 cm bone slab on surface dose with a 220 kVp photon beam, predicting a surface dose increase of up to 3.7% due to bone backscatter while the dose to bone was 210% higher than the surface dose. Butson et al. (2008)^23^ measured 100 kVp x‐rays using an Attix chamber and EBT GAFCHROMIC film and found the maximum dose (D_max_) increased by 12.5% when a 1 cm bone layer is within 0.5 mm from the surface, but lowered to 5% when the same bone layer was 5 cm deep. Baines et al. (2018)^24^ combined both measurements using an Advanced Markus chamber and predicted values from EGSnrc to study dose effects with underlying air or bone, thereby confirming the MC results with physical measurements. Komanduri et al. (1996)^25^ compared dose distributions between superficial x‐rays and electron beams using ITS Monte Carlo code, showing that x‐rays can lead to higher doses to bone due to the photoelectric effect's dominance at lower energies.

Our study builds on these methodologies by integrating the well‐validated EGSnrc (Electron Gamma Shower) code developed by the National Research Council of Canada,^26^ integrated into the SmART‐Plan small animal treatment planning system^27^ at superficial energies (<100 kVp) to conveniently model specific patient anatomies, focusing on the impact of bone at various depths and beam qualities. We enhance the accuracy of bone dose calculations by employing smaller voxel sizes (high‐resolution) MC simulated phantoms, offering a more detailed understanding of spatial dose predictions.

## 2 MATERIALS AND METHODS

### 2.1 The superficial x‐ray machine unit

The SRT‐100 (Sensus Healthcare, Florida, USA) is designed to deliver x‐rays with three treatment techniques: (a) 100 kV at 8 mA, 2.10 mm aluminum half‐value leyer (HVL), (b) 70 kV at 10 mA, 1.10 mm aluminum HVL, and (c) 50 kV at 10 mA, 0.45 mm aluminum HVL. The x‐ray output is 600 cGy at 15 cm source to skin distance (SSD)/100 kV. Table S1 shows the three treatment technique parameters. The aluminum filter changes automatically based on the selected kV and mA. The SRT‐100 machine comes with various sizes of applicators (see Figure S1 and Table S2 for the dimensions of the applicators). The x‐ray tube on the SRT‐100 machine has a tungsten target, a built‐in 0.8 ± 0.1 mm beryllium filter, and a 5.5 mm focal spot. The vendor provided the necessary SRT‐100 specifications.

### 2.2 EGSnrc simulations of the SRT‐100 x‐ray machine

**FIGURE 1:**
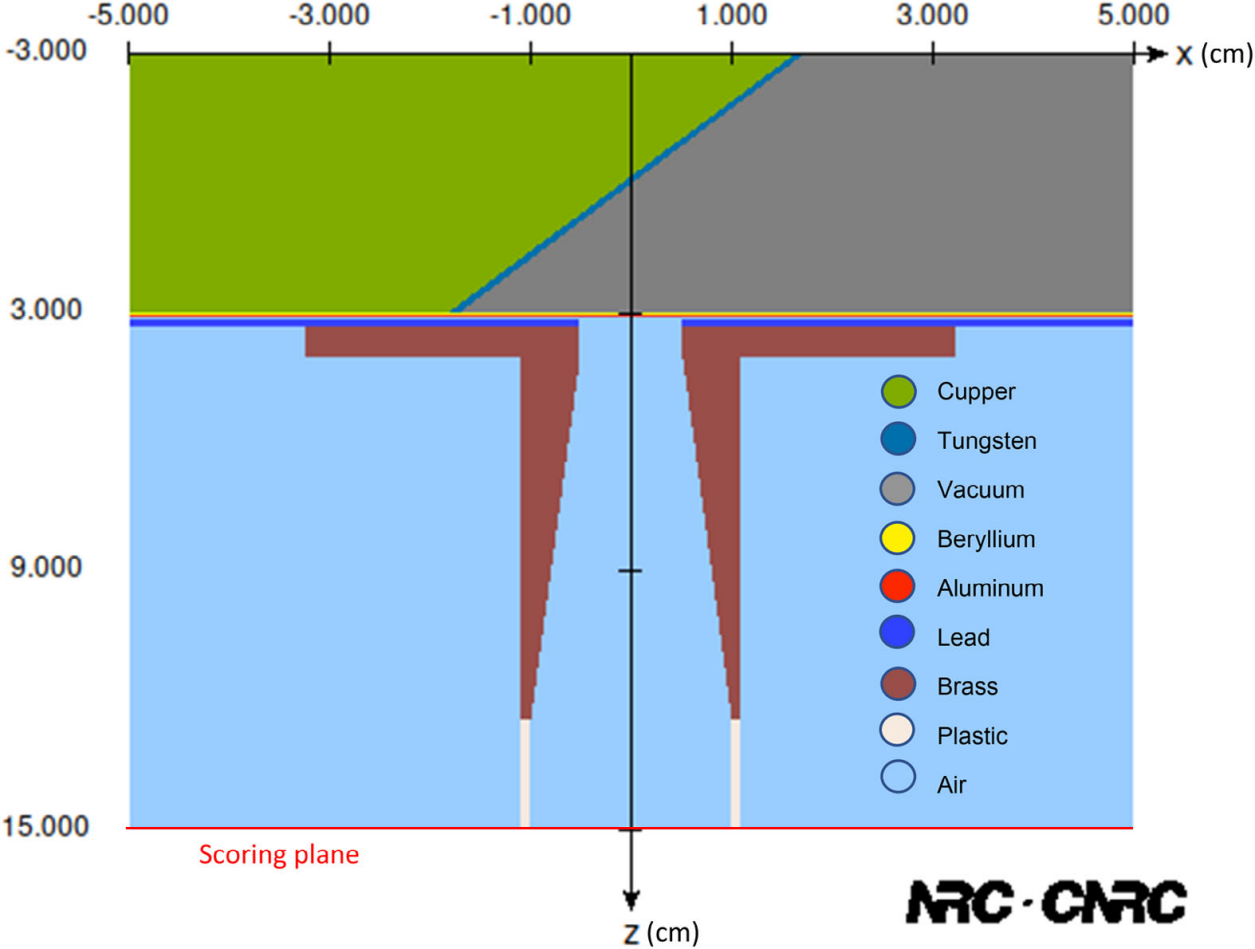
A scheme of the simulated Sensus SRT‐100 treatment head with the 2 cm diameter applicator.

The three treatment energies in the SRT‐100 were simulated using BEAMnrc, a package that models radiation beams through geometrical materials.^28^ The simulated SRT‐100 x‐ray machine with the 2 cm diameter applicator is shown in Figure 1.

**FIGURE 2:**
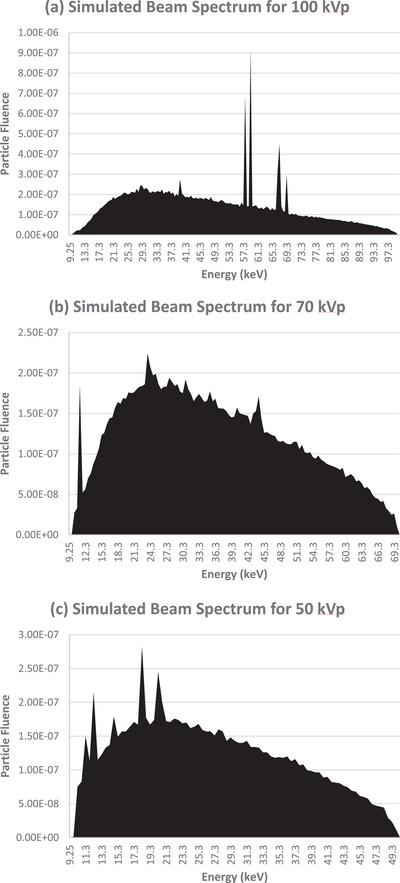
Particle fluence spectra generated by the simulation of (a) 100 kVp, (b) 70 kVp, and (c) 50 kVp treatment techniques.

The simulations were run with 10^8^ number of histories on a computer occupied with AMD Ryzen 9 5900 × 12‐core, 24‐thread CPU. BEAMnrc then generated a phase space file, a file that has the position, direction, and energy of each particle when crossing a specific plane scored at the exit plane of the applicator at 15 cm SSD. The energy spectrum of the three energies (100, 70, and 50 kVp) is shown in Figure 2, utilizing the BEAMdp package.^29^ The Monte Carlo physics parameters that were implemented in the simulations are outlined in Table 1.

**TABLE 1:** Monte Carlo physics parameters.

Maximum step size (cm)	1e10
Max. fractional energy loss/step	0.1
Ximax	0.5
Boundary crossing algorithm	EXACT
Skin depth for BCA	Default
Electron‐step algorithm what	PRESTA‐A‐II
Spin effect	On
Electron impact ionization	On
Brems angular sampling	KM
Brems cross‐sections	NRC
Bound Compton scattering	Norej
Compton cross‐sections	Default
Photoelectron angular sampling	On
Rayleigh scattering	On
Atomic relaxations	EADL
Photon cross‐sections	Xcom
Bremsstrahlung splitting	Directional: 5000 Brem splitting number (for all energies)
Brems cross‐section enhancement	On: 500 enhancement constant for 50, 100 enhancement constant for 70 and 100 kVp.
Global electron cut‐off energy—ECUT (MeV)	0.521
Global photon cut‐off energy—ECUT (MeV)	0.01
Electron range rejection	Off
Photon forcing	Off

To further validate the quality of the simulated beam, the HVL for each energy beam was calculated from the energy distribution within the entire field (except near the applicator walls) that was extracted from the phase space data. To determine the thickness of the material (denoted as “*t*”) required to reduce the air KERMA to half its original value with no absorber *k*(0), the following equation was employed^30^:

$$\begin{array}{ccc} & & k\left(t\right)/k\left(0\right)=\\ & & \;\;\left(\sum_{i=1}^{N}\varphi_{i}E_{i}{\left\{\frac{\mu_{\mathrm{en}}}{\rho }\left(E_{i}\right)\right\}}_{\mathrm{air}}\cdot \exp\left[-\mu {\left(E_{i}\right)}_{\mathrm{absorber}}t\right]\Delta E_{i}\right)\\ & & \;\cdot \;\;{\left(\sum_{i=1}^{N}\varphi_{i}E_{i}{\left\{\frac{\mu_{\mathrm{en}}}{\rho }\left(E_{i}\right)\right\}}_{\mathrm{air}}\Delta E_{i}\right)}^{-1}=0.5 \end{array}$$

The summation was carried out across *N* energy bins, each having a width ∆*E* *_i_* and centered at *E_i_*, representing the midpoint energy. The particle fluence within each energy bin, denoted as *ϕ_i_*, was obtained from the phase space files using the BEAMdp utility program. To generate the energy‐dependent attenuation coefficients (µ) and mass absorption coefficients (µen/ρ), The program “g” was utilized to generate the energy‐dependent attenuation coefficients (*μ*) and mass absorption coefficients (*µ*_en_/*ρ*).^33^ The resulted HVLs are shown in Table 2.

**TABLE 2:** HVLs from simulation, manufacturer, and measurements.

Energy (kVp)	Manufacturer HVL (mm Al)	Measurement HVL (mm Al)	Simulation HVL (mm Al)	Percent difference simulation and manufacturer HVL/simulation and measurement HVL (%)
50	0.45	0.483	0.52	14.4/7.4
70	1.10	1.192	0.97	12.5/20.5
100	2.10	2.074	2.10	0.0/1.2

### 2.3 DOSXYZnrc water phantom simulation

The phase space file was then used as a source to predict deposited dose in a 30 × 30 × 10 cm^3^ water phantom (following the AAPM's TG‐61 protocol for kilovoltage x‐ray beam dosimetry) with a voxel size of 0.5 × 0.5 × 0.05 cm^3^. The water phantom irradiation was simulated using the DOSXYZnrc package.^32^ We assigned the H20521ICRU material for water throughout this study (the cross‐section data for the materials used in the simulation are produced by PEGS4, the preprocessor that creates cross‐section data sets for EGSnrc). The percent depth dose (PDD) along the central axis was then calculated for the three energies (100, 70, and 50 kVp). The generated PDDs were compared with the PDDs calculated from laboratory measurements. To extract and visualize the PDDs, a Monte Carlo dose website viewer (VICTORIA) was utilized.^33^

### 2.4 DOSXYZnrc water–bone slabs phantom simulation

A water–bone slabs phantom with dimensions 10 × 10 × 3 cm^3^ and 0.5 × 0.5 × 0.05 cm^3^ voxel size, made of five slabs total, was simulated using the DOSXYZnrc package. The thickness of each slab is 0.5 cm except for the last water slab, that is 1 cm thick. We assigned the ICRPBONE521ICRU material for bone throughout this study. The PDDs along the central axis of the water–bone phantom for the three x‐ray energies were calculated. Figure S2 shows a 3D printed water–bone phantom that was simulated.

### 2.5 Laboratory measurements of PDDs

**FIGURE 3:**
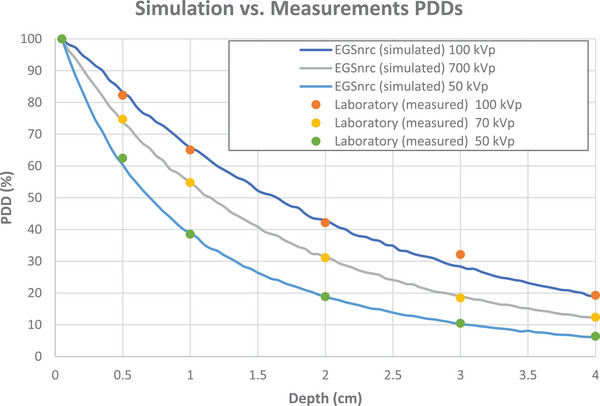
The compared PDDs (normalized to surface dose) from the simulation and the laboratory measurements. (a) 100 kVp, (b) 70 kVp, and (c) 50 kVp.

The PDDs of the Sensus SRT‐100 machine were measured by irradiating a 30 × 30 × 30 cm^3^ water tank at room temperature (25°C) at different depths (5, 10, 20, 30, and 40 mm). The dose was scored using an ion chamber (PTW 30013 0.6 cm^3^, serial number 1812), calibrated to Cobalt‐60 at 5.385 × 10^7^ Gy/C. The charge from the ion chamber was then measured in an electrometer bias (300 V Standard Imaging Max 4000 plus). The PDDs were then calculated and compared to the PPDs from the EGSnrc simulations (Figure 3).

### 2.6 Transmission simulations

**FIGURE 4:**
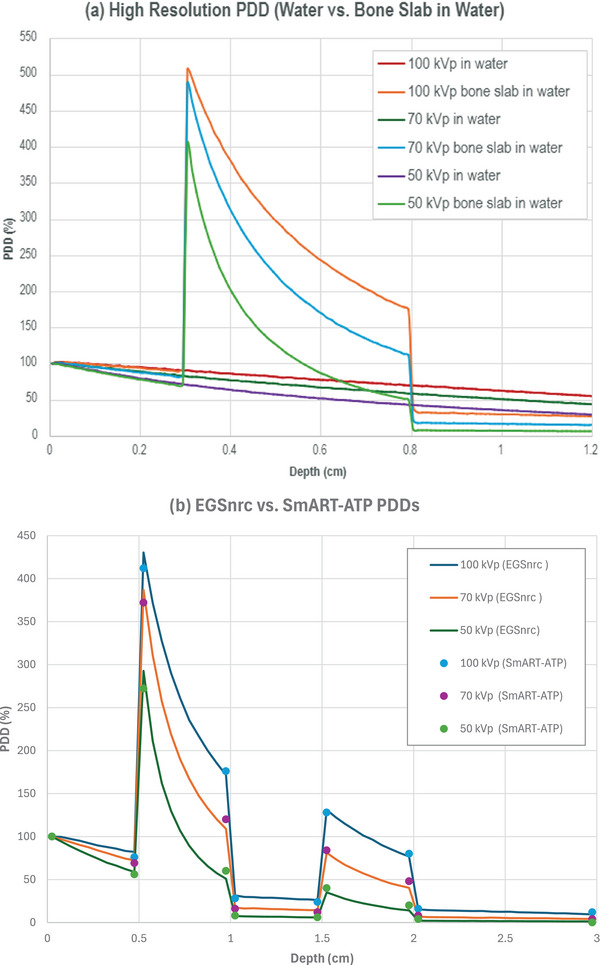
(a) PDDs (normalized to surface dose) of water phantom against PDDs of a water–bone phantom with a 0.5 cm slab of bone at 0.3 cm depth for all three energies, using 0.01 cm voxel size along the z‐axis. (b) SmART‐ATP versus EGSnrc PDDs (normalized to surface dose) at the central axis of the water–bone slabs phantom, using 0.05 cm voxel size along the *z*‐axis.

To indirectly predict the dose deposited in bone, and due to the lack of having a dosimeter that measures the dose deposited in bone, we simulated two water phantoms, both with dimensions of 3 × 3 × 1.28 cm^3^ and a voxel size of 0.5 × 0.5 × 0.01 cm^3^. One phantom contained a 0.5 cm thick bone slab at a depth of 0.3 cm, while the other consisted only of water. We compared the dose in water at a depth of 0.9 cm below the bone slab between the two phantoms (see Figure 4a). To get a more accurate spatial dose distribution, we used a small voxel size along the *z*‐axis (0.01 cm) and increased the number of histories to 10^9^ to overcome the loss of signal‐to‐noise ratio (SNR). We kept the transmission phantom relatively small to save MC computational time that is caused by the small voxel size, as we found no significant difference in predicted dose with varying phantom sizes.

### 2.7 Utilizing EGSnrc phase space file in commercial treatment planning system

The EGSnrc phase space files were then inserted in the SmART‐ATP treatment planning system (SmART Scientific Solutions, Maastricht, the Netherlands)‐ATP for small animal radiotherapy planning. The phase space files of the three energies (100, 70, and 50 kVp) were used to irradiate an acquired cone‐beam computed tomography (CBCT) image of 3D printed water–bone slabs phantom with 0.02 × 0.02 × 0.02 cm^3^ voxel size. We then compared the PDD from the EGSnrc modeled water–bone phantom (from Section 2.4) to the PDD from SmART‐ATP of the CBCT image of the 3D printed phantom (Figure 4b).

### 2.8 Utilizing EGSnrc simulated beams in clinical DICOM image for treatment planning

**FIGURE 5:**
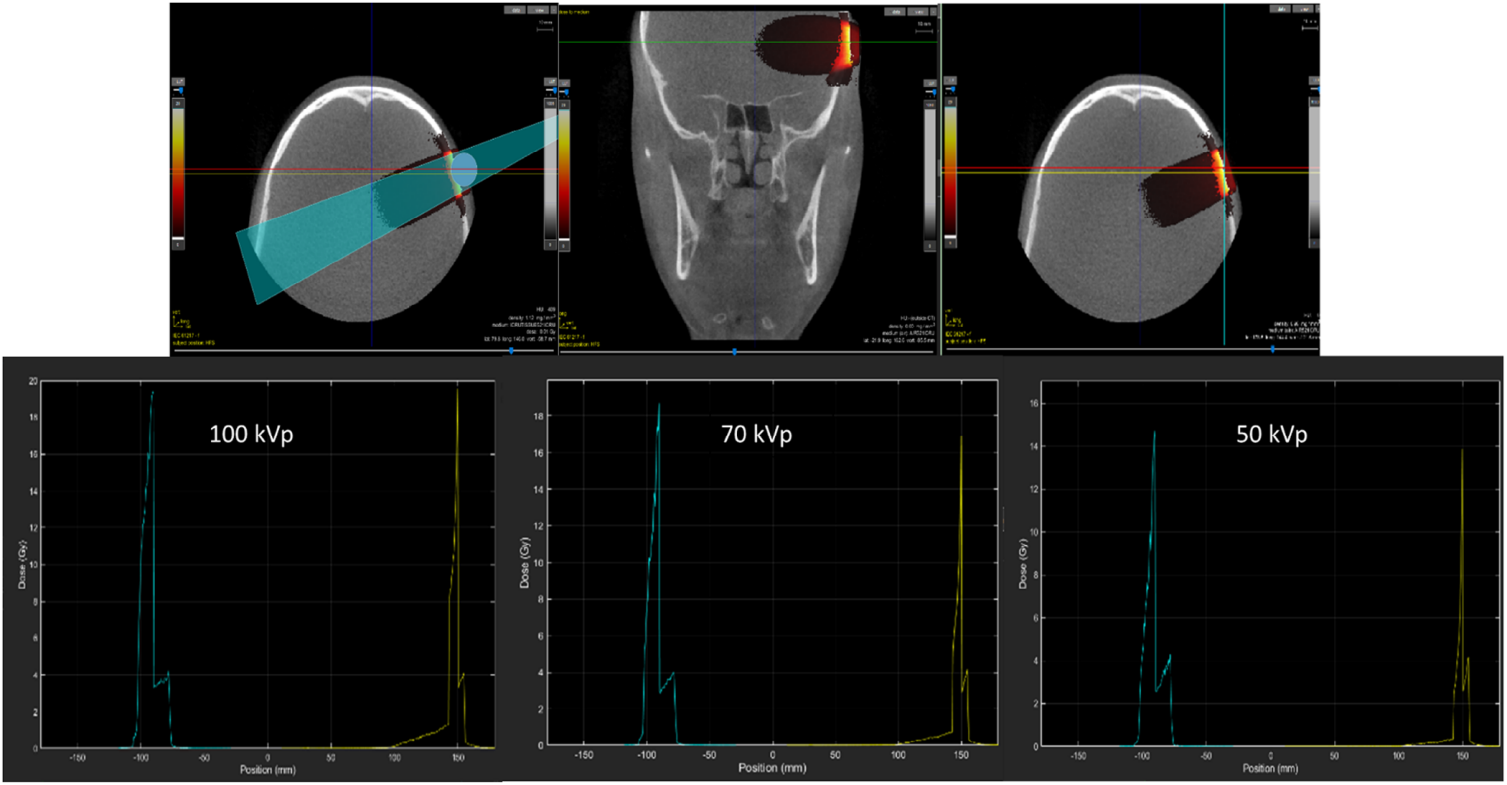
(Top left) A treatment plan for a 100 kV simulated x‐ray beam from Sensus SRT‐100 for a human head CBCT scan. The color wash corresponds to the dose distribution, where white/yellow is the highest dose. The blue circle is the planned isocenter, and the blue cone is the planned radiation beam. The treatment plan was applied to the same CBCT scan with three energies (50, 70, and 100 kVp). (Top middle and top right) A line profile of the dose deposited along the line was drawn, and three curves were generated for the three energies (three bottom figures). The yellow curve corresponds to the yellow horizontal line on the bottom right image. The cyan curve corresponds to the vertical cyan line on the bottom right image. The peak that is seen on the cyan and yellow curves is a result of the energy deposited in the bone from the human's skull. The isocenter with three different beam energies (100, 70, and 50 kVp) is receiving 2 Gy total. CBCT, cone‐beam computed tomography.

Finally, a Digital Imaging and Communications in Medicine (DICOM) file of a human head CBCT was imported to apply the phase space file as a beam source for a treatment plan, utilizing the three energy beams from the Sensus SRT‐100 machine (Figure 5).

### 2.9 Statistics

Statistical analysis was performed using GraphPad Prism version 10.0 (GraphPad Software, San Diego, California, USA). Root mean square error (RMSE) was calculated to compare the simulated PDD curves to laboratory measurements. The statistical error of the MC simulations was estimated based on the standard deviation of the mean energy deposited in scoring voxels. Dose ratios at water–bone interfaces were calculated by dividing the dose in bone by the dose in water at the interface. For transmission simulations comparing phantoms with and without bone, the ratio of doses at 0.9 cm depth was calculated. All simulations were run with at least 10^8^ particle histories to ensure statistical certainty.

## 3 RESULTS

### 3.1 Monte Carlo predictions versus laboratory measurement PDDs

EGSnrc was able to simulate the 100, 70, and 50 kVp with RMSE of percentage depth dose ratios between Monte Carlo predictions and lab measurements of 1.66, 0.47, and 0.99, respectively. The statistical error of the simulations with 0.05 cm voxel size is 1% and goes down to 0.6% with 0.01 cm voxel size. The ratios of the PDDs in the water–bone interfaces for EGSnrc and SmART‐ATP are listed in Table S3. Table S4 shows the ratios of the PDDs in the water–bone interfaces for EGSnrc with a 0.01 cm voxel size along the *z*‐axis.

### 3.2 Dose to bone from superficial x‐ray energies

**FIGURE 6:**
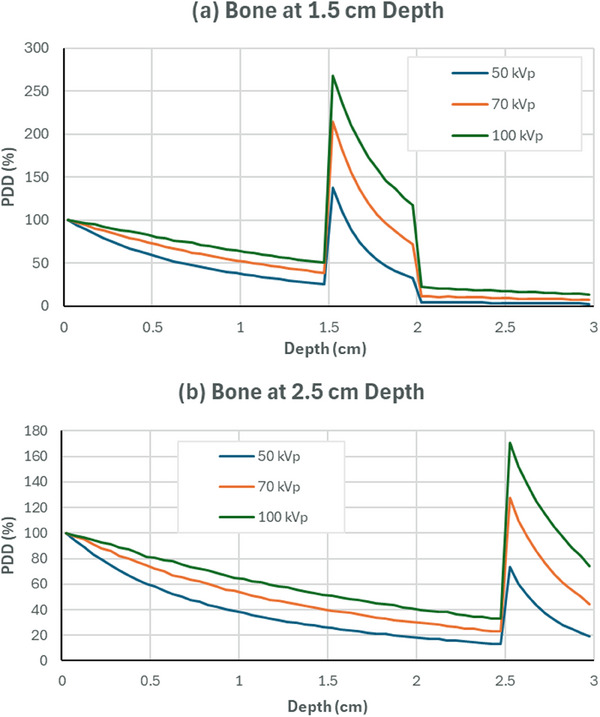
PDD curves from Monte Carlo simulations of a water phantom with a bone slab located at (a) 1.5 cm depth and (b) 2.5 cm depth. The curves illustrate the dose buildup phenomenon at the bone–water interface for different incident beam energies (50, 70, and 100 kVp).

The dose to bone at a depth of 0.3 cm is about 510% higher than the dose to water at the surface for the 100 kVp, 490% higher for the 70 kVp, and 410% higher for the 50 kVp. Furthermore, Figure 6 shows the PDD curves obtained from MC simulated water phantom with a 0.5 cm bone slab at different depths. In the left‐hand image, the bone slab is placed at a depth of 1.5 cm that predicts a dose to bone reaching approximately 270% for the 100 kVp beam, 215% for the 70 kVp beam, and 140% for the 50 kVp beam, relative to the surface dose. In the right‐hand image, the bone slab is placed at a deeper depth of 2.5 cm. The magnitude of bone dose enhancement is around 74% for the 50 kVp beam, 130% for the 70 kVp beam, and 170% for the 100 kVp beam, relative to the surface dose. Based on our transmission simulations, the ratios of the dose attenuated in bone and water at 0.9 cm depth are 2.12, 3.13, and 5.27 with 100, 70, and 50 kVp, respectively.

The enhanced dose to bone can be observed by running the simulated beams of the three energies in the SmART‐ATP treatment system using a DICOM image of a human head CBCT. The surface dose in three cases was 4 Gy and increased at the bone interface (the human skull) to around 20, 19, and 15 Gy with the 100, 70, and 50 kVp beams, respectively.

## 4 DISCUSSION

In this study, EGSnrc accurately modeled the superficial radiation therapy machine for the three energies. EGSnrc has a proven efficiency in modeling superficial and orthovoltage x‐rays for radiation therapy units, dosimetry, and treatment planning.^34^, ^35^

There are a few important points to consider regarding the PDD simulations. The PDD curves change with a change in the voxel size of the water phantom along the *z*‐axis. We found that the most accurate simulation results were reached by setting the voxel size to 0.05 cm along the *z*‐axis. The choice of voxel size in MC simulations for radiotherapy treatment planning involves a trade‐off between spatial resolution, statistical uncertainty, and computational time. Smaller voxels provide finer spatial detail, which is particularly important for complex radiotherapy treatments. Additionally, a smaller voxel size significantly increases computational time.^36^ However, smaller voxels result in fewer particles per voxel, potentially leading to higher statistical noise.^37^

When using an ion chamber, such as the PTW 30013 (0.6 cm^3^), to measure the PDD within a water phantom, it is generally not advisable to measure the dose right at the surface of the phantom. The reason is that the dose would vary significantly over a very shallow depth range at the surface. That is, due to two primary factors: electron contamination coming from the treatment head and increased electron scatter arising from the air‐water interface.^38^, ^39^ For that reason, the ratio between the next known depth and the surface was obtained from the simulated PDD curve.

From the spectra generated by the BEAMdp toolkit, we notice that the 70 kVp spectrum does not show prominent characteristic x‐ray peaks. Tungsten has several characteristic x‐ray lines, with energies around 59.3 keV (Kα1), 67.2 keV (Kα2), and 68.8 keV (Kβ). However, these energies are relatively high. Characteristic x‐rays are indeed produced at 70 kVp, but they are outweighed by the more intense Bremsstrahlung radiation due to the energy limitations of the electron beam at this voltage.

EGSnrc and SmART‐ATP MC predictions demonstrated that the dose to bone from superficial x‐ray energies is four to five times higher than the dose to water at the surface. That is, due to the domination of the photoelectric effect interaction of low energy x‐rays with bone. With higher energy beams (70–100 kVp) generally resulting in a more pronounced dose buildup peak due to their increased penetration capability and production of secondary electrons.

## 5 FUTURE DIRECTIONS

Since measuring the dose right at the surface of the water phantom with an ionization chamber is not advisable due to electron contamination and scatter effects, future research might explore alternative dosimetry techniques or develop correction factors to account for these effects in surface dose measurements.^40^, ^41^

Building on the accurate modeling of superficial and orthovoltage x‐rays, future research could explore the clinical applications of these findings. Bone density varies across individuals due to factors like age, genetics, and health conditions.^42^ Studies have shown that the anatomy of each patient, including the position of bone relative to the skin surface, can significantly change the radiation dose distribution. Our study shows that the depth of bone influences the dose at bone‐soft tissue interfaces, with shallower bones leading to higher dose peaks relative to the surface dose. For treatments near bony structures, understanding individual bone anatomy and density is crucial. Personalized treatment plans could incorporate detailed anatomical and density information from computed tomography (CT) scans or magnetic resonance imaging (MRI), and even more bone density and atomic information from dual‐energy x‐ray absorptiometry (DEXA) scans.^43^ The material properties can then then imported into MC treatment planning systems where clinicians can simulate and visualize the radiation dose in specific patient anatomies to predict and adjust for dose enhancement, potentially preventing overexposure that could lead to bone necrosis, fracture, or other complications.^44^ We believe understanding accurate dose absorption within the tissue bone interfaces allows for the optimization of beam angles, number of beams, and energy selection to minimize dose to normal bone. Future work could explore the integration of DICOM image cases for bone tumor IORT procedures. Additionally, further research could investigate the biological effects of higher dose deposition in bone by superficial x‐rays using bone cell cultures and comparing their cell survival with the survival of cancerous bone cells.

## 6 CONCUSIONS

MC simulations effectively quantified the significant bone dose enhancement during radiotherapy with superficial x‐rays, highlighting the importance of accounting for this effect in treatment planning and dose calculations. Additionally, clinicians should consider measuring the depth of bone below the surface prior to treatment of tumors to avoid excessive bone dose.

## AUTHOR CONTRIBUTIONS

All listed authors contributed to the conception or design of the work; or the acquisition, analysis, or interpretation of data for the work; drafted the work or revised it critically for important intellectual content; final approval of the version to be published; and agreed to be accountable for all aspects of the work.

## CONFLICT OF INTEREST STATEMENT

The SRT‐100 superficial x‐ray unit was lent by Sensus Healthcare to CSU for scientific and clinical evaluation. The unit is currently still at CSU. The engineering diagrams for the x‐ray source as well as the material properties were also provided by Sensus. Importantly, the relationship between CSU and Sensus Healthcare was in no way contingent on the nature results of this manuscript nor the interpretation within the Discussion. The study would have been conducted in the same unbiased manner whether the unit was on loan, donated, or purchased from Sensus Healthcare.

## Supporting information



Supporting information

Supporting information

## Data Availability

Authors will share data upon request to the corresponding author.
