# Supplementary material for: Monte Carlo modeling of radiation dose from radiation therapy with superficial x‐rays
Source: J Appl Clin Med Phys. 2025 Mar 4;26(6):e70062. doi: 10.1002/acm2.70062 (PMC12148772; doi:10.1002/acm2.70062)

Appendix Figures Captions

Figure A1. The Sensus SRT-100 commonly used applicators.

Figure A2. A 3D printed 3x3 cm^2 water-bone slabs phantom. The red slabs are equivalent to soft tissue (1.1 g/cm3, ~100 HU @ 90 kVp) and the grey slabs are equivalent to bone (1.48 g/cm3, ~1225 HU @90 kVp).

Figure A1.

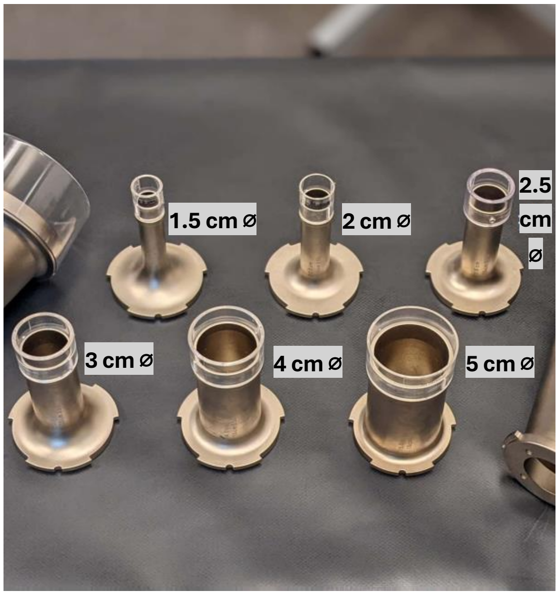

Figure A2.

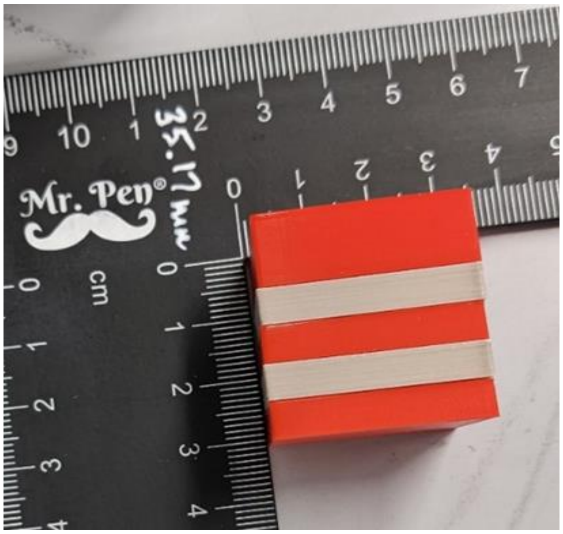

Supplement: Supplementary file 1 — Supporting information [file ACM2-26-e70062-s002.pdf]
