# Supplementary material for: Monte Carlo modeling of radiation dose from radiation therapy with superficial x‐rays
Source: J Appl Clin Med Phys. 2025 Mar 4;26(6):e70062. doi: 10.1002/acm2.70062 (PMC12148772; doi:10.1002/acm2.70062)
Supplement: Supplementary file 2 — Supporting information [file ACM2-26-e70062-s001.docx]

Table A1. SRT-100 treatment technique parameters.

| X-ray kVp | HVL (mm Al) | Tube Current (mA) |
| --- | --- | --- |
| 100 | 2.10 | 8 |
| 70 | 1.10 | 10 |
| 50 | 0.45 | 10 |

*HVL: Half-value layer

Table A2. the dimensions of the applicators that were used in this study.

| Applicator Number | Applicator Diameter (cm) | Source to Surface Distance (cm) |
| --- | --- | --- |
| #1 | 1.5 | 15 |
| #2 | 2.0 | 15 |
| #3 | 2.5 | 15 |
| #4 | 3.0 | 15 |
| #5 | 4.0 | 15 |
| #6 | 5.0 | 15 |

Table A3. The ratios of the doses in the water-bone interfaces for EGSnrc and SmART-ATP

| Monte Carlo method/Energy (kVp) | EGSnrc/100 | EGSnrc/70 | EGSnrc/50 | SmART-ATP/100 | SmART-ATP/70 | SmART-ATP/50 |
| --- | --- | --- | --- | --- | --- | --- |
| water/bone interface | 5.19 | 5.32 | 4.94 | 5.42 | 5.38 | 4.86 |

Table A4. The ratios of the PDDs in the water-bone interfaces for EGSnrc with 0.01 cm voxel size along the z-axis.

| Energy (kVp) | 100 | 70 | 50 |
| --- | --- | --- | --- |
| water/bone interface | 5.37 | 5.69 | 5.74 |
